# A Rapid and Efficient Method for Isolation and Transformation of Cotton Callus Protoplast

**DOI:** 10.3390/ijms23158368

**Published:** 2022-07-28

**Authors:** Peilin Wang, Yuanchun Pu, Muhammad Ali Abid, Linglin Kang, Yulu Ye, Man Zhang, Chengzhen Liang, Yunxiao Wei, Rui Zhang, Zhigang Meng

**Affiliations:** 1 Biotechnology Research Institute, Chinese Academy of Agricultural Sciences, Beijing 100081, China; wangpeilin19@126.com (P.W.); puyuanchun1209@126.com (Y.P.); abid@caas.cn (M.A.A.); kanglinglin@126.com (L.K.); yulu_ye16@163.com (Y.Y.); mzhang0825@163.com (M.Z.); liangchengzhen@caas.cn (C.L.); weiyunxiao@caas.cn (Y.W.); 2 College of Agronomy, Xinjiang Agricultural University, Urumqi 830052, China

**Keywords:** protoplasts, cotton, transformation efficiency, enzyme

## Abstract

Protoplasts, which lack cell walls, are ideal research materials for genetic engineering. They are commonly employed in fusion (they can be used for more distant somatic cell fusion to obtain somatic hybrids), genetic transformation, plant regeneration, and other applications. Cotton is grown throughout the world and is the most economically important crop globally. It is therefore critical to study successful extraction and transformation efficiency of cotton protoplasts. In the present study, a cotton callus protoplast extraction method was tested to optimize the ratio of enzymes (cellulase, pectinase, macerozyme R-10, and hemicellulase) used in the procedure. The optimized ratio significantly increased the quantity and activity of protoplasts extracted. We showed that when enzyme concentrations of 1.5% cellulase and 1.5% pectinase, and either 1.5% or 0.5% macerozyme and 0.5% hemicellulase were used, one can obtain increasingly stable protoplasts. We successfully obtained fluorescent protoplasts by transiently expressing fluorescent proteins in the isolated protoplasts. The protoplasts were determined to be suitable for use in further experimental studies. We also studied the influence of plasmid concentration and transformation time on protoplast transformation efficiency. When the plasmid concentration reaches 16 µg and the transformation time is controlled within 12–16 h, the best transformation efficiency can be obtained. In summary, this study presents efficient extraction and transformation techniques for cotton protoplasts.

## 1. Introduction

Methods for functional genomics research have developed rapidly in recent years, and researchers can now clone and functionally analyze new genes using various molecular biology techniques. Researchers commonly determine the biological function of a gene by overexpressing or knocking out the gene. However, stable genetic transformation in common crops requires a great deal of time and effort, usually requiring at least three months in *Oryza sativa* [1], eight–ten months in maize [2], and more than a year in cotton [3]. Transient expression platforms have the potential to break through this bottleneck [4]. As the name indicates, transient expression platforms rely on short-term expression of transgenes in a species of interest without integration of transgenic cassettes into the host genome. An ideal transient expression platform should be technologically simple, quick, low-cost, robust, high-throughput, and have high transformation efficiency. It should also provide a means of assessing the efficiency of expression. Many transient expression platforms for crop species currently rely on the soil bacterium *Agrobacterium tumefaciens* to deliver expression cassettes into plant nuclei via transfer (T)-DNA [5,6,7]. Although these methods can provide suitable levels of transgene expression in planta, they are low-throughput and often require mature plant tissue, increasing the time and growth space required to acquire data. Moreover, infection by *A. tumefaciens* can induce widespread changes in gene expression in the host, which may influence the dynamics of transgene expression [8]. Another approach is the bombardment of plant tissue with DNA-carrying microprojectiles [9,10], but such procedures require sophisticated equipment, are low-throughput, and can be expensive due to the high cost of DNA microcarriers. Plant protoplast transformation has also been widely used, and this method offers many advantages over other techniques. It does not require sophisticated equipment, allows for high-throughput screening, and is relatively inexpensive, quick, and robust. In addition, protoplasts are routinely isolated from young tissues (7–10-day-old seedlings), reducing the required growth space and time. In vivo transient protoplast transfection is an effective tool for studying gene expression, metabolic pathways, and molecular biology. Although protoplasts can be isolated from cell suspension culture or germinating seedlings, preparation of those donor tissues can be less efficient, and the process is usually time-consuming and laborious. Protoplasts have been critical for studying many aspects of plant biology, including hybridization, chloroplasts, and plant defense mechanisms [11]. Protoplast-based transient expression assays are convenient because they allow for rapid and high-throughput analysis of gene expression, subcellular localization, protein activity, and protein–protein interactions [12]. Transient expression systems have been used in rice, *Panicum virgatum* L. [13], barley [14], grapevine [11], wheat [15], ryegrass [16], and Arabidopsis [17,18].

Cotton is one of the most economically important crops worldwide, providing natural fibers to textile industries around the globe [19]. Although cotton fiber is the most important raw material in the world for textiles, its complex genome and specialized metabolites have presented challenges in molecular biology research [20]. At present, the pollen tube pathway and Agrobacterium-mediated transformation are the most commonly used methods for cotton genetic transformation, but the transformation efficiency of both is very low [3]. Protoplast isolation is more challenging in cotton than in other species due to the high levels of polysaccharide and polyphenol characteristics. In the majority of molecular biology experiments involving cotton, Arabidopsis thaliana protoplasts are substituted for cotton protoplasts despite their vastly different genetic origins.

Due to the high research value of cotton and the low transformation efficiency, an easy and efficient transient transformation method is urgently needed to overcome research limitations and to facilitate the functional characterization of cotton genes. In this experiment, an extraction protocol was developed in cotton with an optimal ratio of enzyme solutions (cellulase, pectinase, macerozyme, and hemicellulase) to extract cotton callus protoplasts. The principle of single-variable research was used to separately test the concentration of each enzyme while the concentrations of the other three were kept constant. A gradient test was performed, and the number and activity of extracted protoplasts were measured. One combination of enzyme solutions performed significantly better than the others, yielding a suspension of 3.0 × 10^6^ protoplasts per mL with activity above 97%.

## 2. Results

### 2.1. Effects of Different Enzyme Concentrations on Protoplast Extraction

**Figure 1:**
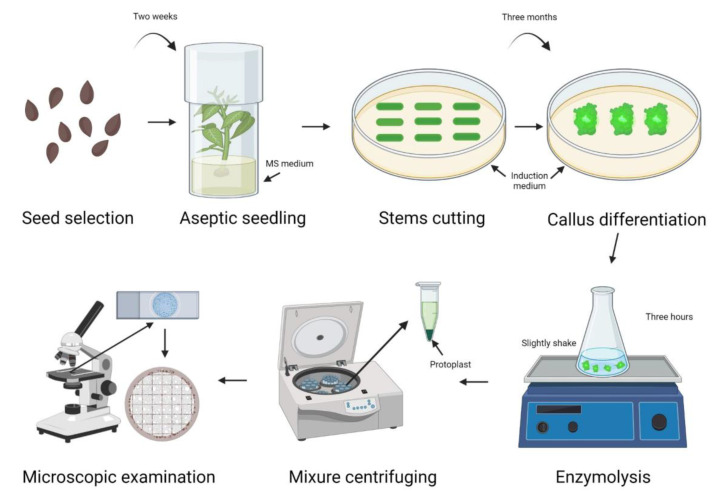
Schematic illustration of protoplast isolation protocol.

In this study, the principle of single-variable experimentation was adopted, keeping the concentration of three enzymes constant and testing a gradient (0%, 0.5%, 1%, and 1.5%) of the fourth (variable) enzyme (Table 1). The experimental protocol is shown in Figure 1. Extracted protoplast number and viability were used to determine the optimal concentration of each enzyme.

**Figure 2:**
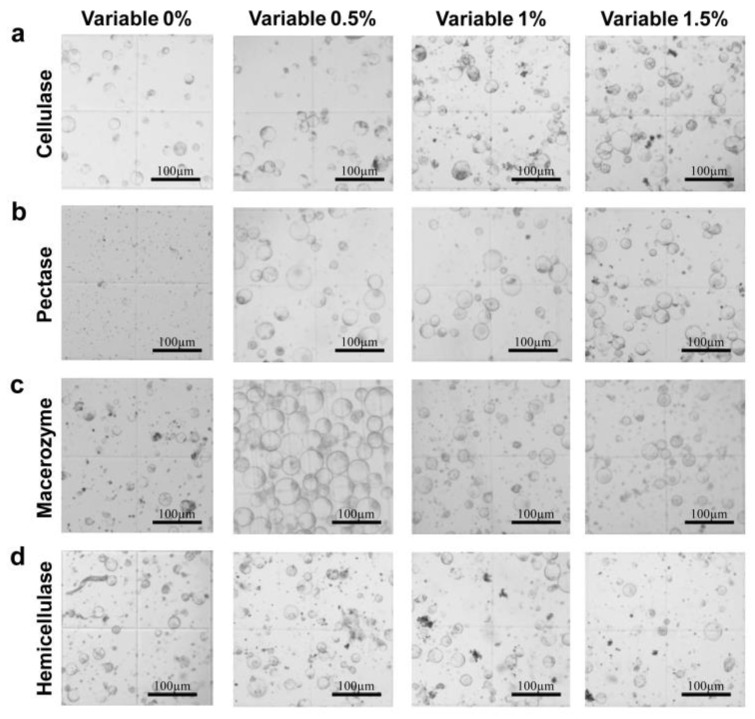
Microscopic examination of protoplasts extracted with solutions containing different concentrations of enzymes. Comparisons of protoplasts extracted with solutions containing different concentrations of (**a**) cellulase, (**b**) pectinase, (**c**) macerozyme, and (**d**) hemicellulase. Scale bars = 100 μm.

**Figure 3:**
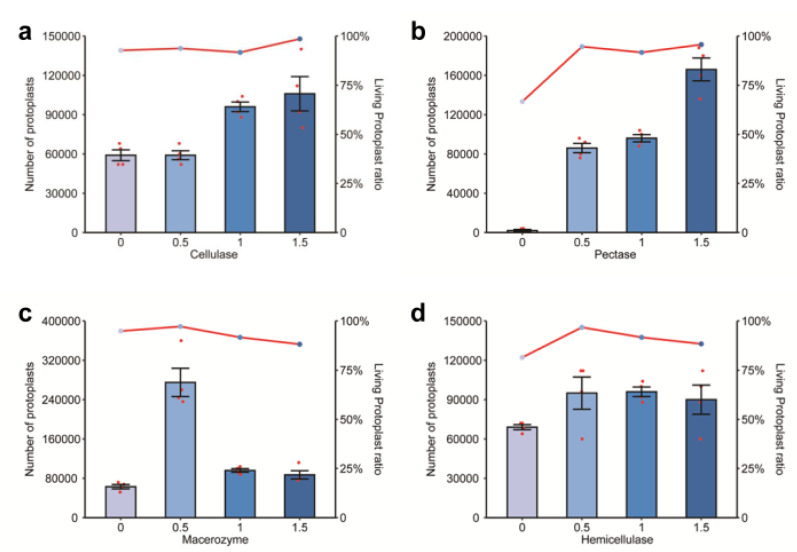
The number and activity of protoplasts extracted with solutions containing different concentrations of enzymes. Graphs show the number and activity of protoplasts isolated with solutions containing different concentrations of (**a**) cellulase, (**b**) pectinase, (**c**) macerozyme, and (**d**) hemicellulase.

The number of protoplasts increased with the increase in cellulase concentration; at 1% cellulase, the number of protoplasts nearly doubled compared to the samples extracted with 0% or 0.5% cellulase. The yield and activity were both highest when the cellulase concentration was 1.5% (Figure 2a and Figure 3a). The optimal protoplast extraction was therefore achieved with 1.5% cellulase.

Very few protoplasts were extracted with 0% pectinase, clearly indicating the importance of pectinase in this extraction. The number of protoplasts increased with the increase in pectinase concentration. There was not a significant difference in the number of protoplasts extracted with 0.5% vs. 1% pectinase, but a concentration of 1.5% pectinase greatly improved the number of protoplasts extracted (Figure 2b). Protoplast activity was comparable at 0.5%, 1%, and 1.5% pectinase (Figure 3b). Therefore, the extraction was best with 1.5% pectinase.

The number of extracted protoplasts was highest when the macerozyme concentration was 0.5%, with nearly three times as many protoplasts extracted compared to the samples extracted with other concentrations of macerozyme (Figure 2c). Protoplast activity was also highest at 0.5% and decreased with increased macerozyme concentration (Figure 3c). Thus, the optimal extraction was achieved with 0.5% macerozyme.

The number of protoplasts extracted was not significantly different among the four concentrations of hemicellulase, but the number of protoplasts was slightly higher at 0.5%, 1%, and 1.5% (Figure 2d). Protoplast activity reached a peak at 0.5% hemicellulase, and the number of living protoplasts decreased with the increase in enzyme concentration (Figure 3d). The extraction effect was therefore optimal when the concentration of hemicellulase was 0.5%.

### 2.2. Effects of Combined Varied Enzyme Concentrations on Protoplasts

**Figure 4:**
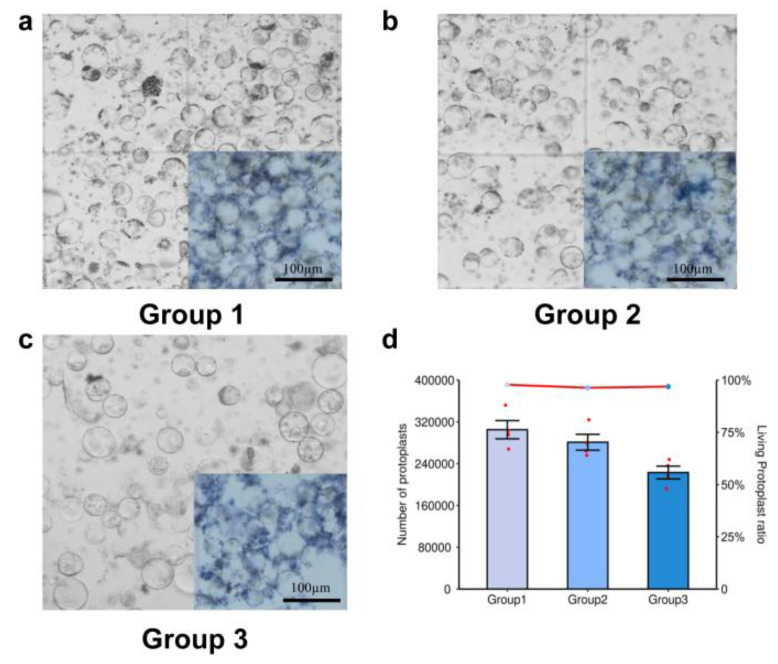
Microscopic examination of protoplasts treated with different concentrations of enzyme solutions. Microscopic examination of protoplasts extracted with (**a**) solution 1: 1.5% cellulase, 1.5% pectinase, 0.5% macerozyme, and 0.5% hemicellulase; (**b**) solution 2: 2.1% cellulase, 2.1% pectinase, 0.7% macerozyme, and 0.7% hemicellulase; and (**c**) solution 3: 0.9% cellulase, 0.9% pectinase, 0.3% macerozyme, and 0.3% hemicellulase. (**d**) Number and activity of protoplasts extracted with different concentrations of enzyme solutions.

Based on the above experiments, we determined that under a constant concentration of the other three enzyme solutions, the optimal concentrations of each enzyme in terms of yield and activity were 1.5% cellulase, 1.5% pectinase, 0.5% macerozyme, and 0.5% hemicellulase. However, it was worth exploring whether extractions could obtain more protoplasts with better activity when enzymes were combined at their optimum concentrations. We therefore designed solutions with different combinations of enzyme concentrations for further experimentation: solution 1 (1.5% cellulase, 1.5% pectinase, 0.5% macerozyme, and 0.5% hemicellulase); solution 2 (2.1% cellulase, 2.1% pectinase, 0.7% macerozyme, and 0.7% hemicellulase); and solution 3 (0.9% cellulase, 0.9% pectinase, 0.3% macerozyme, and 0.3% hemicellulase). The results revealed that the combinatorial effect of the optimal concentration of each individual enzyme was excellent, and the quantity and activity of extracted protoplasts remained high (Figure 4a–c). When the percentage of the total enzyme solution was increased, it was accompanied by impurities, which may have been caused by the high concentration of enzymes (Figure 4b). When the amount of total enzyme was reduced (maintaining the same ratio of each enzyme), impurities were significantly reduced, but the number of protoplasts was also reduced (Figure 4c). Similarly, trypan blue staining showed that there was no significant difference in the concentration of living protoplasts among samples extracted with any of the three solutions (Figure 4d). The lower enzyme concentrations may not have been enough for complete enzymatic hydrolysis. The final enzyme concentrations used were 1.5% cellulase and 1.5% pectinase, and the best results were obtained with either 1.5% or 0.5% macerozyme, and 0.5% hemicellulase.

### 2.3. Protoplast Transformations for Subcellular Localization

**Figure 5:**
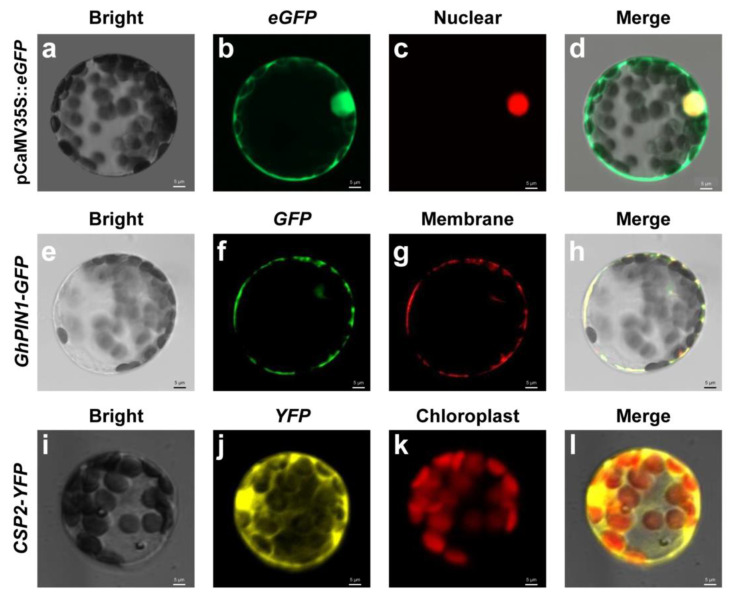
Subcellular localization of marker proteins. Cotton protoplasts transformed with (**a**–**d**) pCaMV35S::*eGFP*, containing mCherry as a nucleus marker; (**e**–**h**) pCaMV35S::*GhPIN1*-*eGFP*, containing mCherry as a plasma membrane marker; and (**i**–**l**) pCaMV35S::*CSP2*-*YFP*, containing mCherry as a chloroplast marker. Scale bars = 5 μm.

Protoplasts are often used to study subcellular localization [21]. To verify whether protoplasts extracted with this method could be used for plasmid transformations, we selected the conventional transient expression vector pCambia1302-CaMV35S::*eGFP* and expressed a nuclear localization signal fused with the mCherry gene as a marker. Protoplasts could constitutively express *eGFP* green fluorescence and nuclear-localized red fluorescence signals, and the two signals were successfully co-expressed (Figure 5a–d). We also selected a membrane-localized gene, *GhPIN1*, for co-expression with *eGFP*. After co-transforming protoplasts, we observed that the membrane-specific expression of *eGFP* and membrane-localized mCherry were successfully co-localized (Figure 5e–h). To test the transformation efficiency of other vectors, we selected the yellow fluorescent protein (YFP) gene vector fused with the *CPS4* gene (pCaMV35S::*CPS4*-*YFP*) and an ER-localized marker. After co-transformation, we observed the *YFP* signal; although *YFP* and mCherry were not co-localized, both were successfully transferred into protoplasts and expressed well (Figure 5i–l). The results showed that the protoplasts extracted using our protocol were active, could be transformed with common vectors, and could transiently express common fluorescent signals. All of these results indicated that the obtained protoplasts can be used for downstream genetic transformation.

### 2.4. Relationship between Plasmid Concentration and Transformation Efficiency

**Figure 6:**
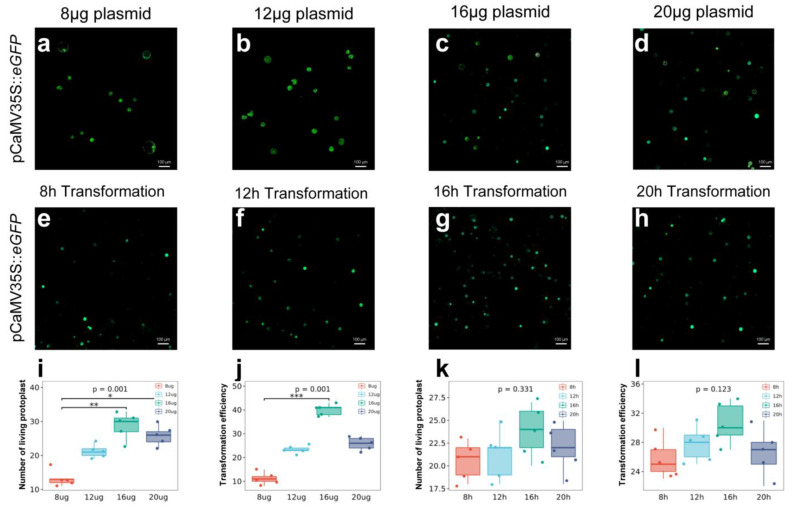
Effects of plasmid concentration and transformation time on protoplast transformation efficiency. Fluorescence microscope images of protoplasts transformed using different (**a**–**d**) plasmid concentrations and (**e**–**h**) transformation times. Scale bars = 100 μm. (**i**) The number of fluorescent protoplasts per unit area after transformations were performed with different plasmid concentrations (*p* = 0.001). (**j**) Protoplast transformation efficiency per unit area after transformations were performed with different plasmid concentrations (*p* = 0.001). (**k**) The number of fluorescent protoplasts per unit area after transformations were performed with different transformation times (*p* = 0.331). (**l**) Protoplast transformation efficiency per unit area after transformations were performed with different transformation times (*p* = 0.123). * *p* < 0.05, ** *p* < 0.01, *** *p* < 0.001 (Dunn’s test).

Many studies have posited that the number of protoplasts and the concentration and conformation of exogenous plasmids affect transformation efficiency. The optimal plasmid concentration is known to differ among plant species and transformation systems. We therefore tested several plasmid concentrations: 8, 12, 16, and 20 µg. Successfully and unsuccessfully transformed protoplasts were measured via microscopy. Notably, the highest number of fluorescent protoplasts was obtained when 16 μg plasmid was used (Figure 6a–d), as quantified by the number of protoplasts obtained (Figure 6i) and the transformation efficiency (Figure 6j). The results of this experiment show that the transformation efficiency increased with higher plasmid concentrations and that the transformation efficiency was highest at 16 µg (35–45%).

### 2.5. Relationship between Transformation Time and Efficiency

We next studied the optimal transformation time for isolated cotton protoplasts. The enzyme solution contained 1.5% cellulase, 1.5% pectinase, 0.5% macerozyme, and 0.5% hemicellulase; 16 µg of plasmid was used as the standard concentration. The transformation times tested were 8, 12, 16, and 20 h. Successfully and unsuccessfully transformed protoplasts were studied via microscopy. The highest number of fluorescent protoplasts was obtained at a transformation time of 16 h (Figure 6e–i,k). There were some differences in the number of fluorescent protoplasts and transformation efficiency among groups, but the differences were not statistically significant. The transformation time had less influence on transformation efficiency than plasmid concentration did. A maximum transformation time of 12–18 h should be used to prevent protoplast rupturing.

## 3. Discussion

Enzymatic hydrolysis is the primary method used for isolating plant protoplasts, and the enzyme types and concentrations are key factors affecting the successful extraction of protoplasts [22]. Enzymes are used in such protocols to degrade cell wall components, such as cellulose, hemicellulose, and pectin, thereby releasing protoplasts. Therefore, different plants require different combinations and concentrations of enzymes depending on the cell wall composition. Cotton is an economically important crop throughout the world. With the release of cotton genome sequence information, functional genomics methods have developed rapidly in cotton, and many functional genes have been discovered. However, the biological characteristics of a number of important cotton genes remain to be determined. To date, there have been few reports involving functional verification of target genes using transformed cotton protoplasts. Because cotton contains a high concentration of polysaccharides and polyphenols, it is relatively difficult to perform nucleic acid, protein, and organelle extractions. Therefore, the components and working concentrations of the enzymatic hydrolysis solution are particularly important.

The type and concentration of enzymes should be selected according to the source of plant material and physiological state, and the appropriate combination of enzyme solution should be selected for different explant materials [23]. The separation of cotton protoplasts basically adopts the method of enzymatic hydrolysis; in one study, a combination of 4.0% cellulase and 0.4% pectinase was used to separate cotton cotyledon mesophyll protoplasts [24]. Another used 3.0% cellulase, 0.5% hemicellulase, 1.5% pectinase, and enzymatic hydrolysis for 20 h to isolate protoplasts from six different explants [25]. Another work used cotton cotyledons as explants, using a combination of 1.5% cellulase and 0.4% macerozyme, and enzymatic hydrolysis for 3–12 h; they successfully isolated cotton protoplasts and performed gene transient expression analysis [26]. Other researchers obtained cotton protoplasts with a yield of 1.0 × 10^6^ mL^−1^ and an activity of 90% by extracting the protoplasts of cotton cotyledons; they used a combination of 1.5% cellulase and 0.4% macerozyme solution, but did not discuss transformation efficiency [27]. Although the isolation of protoplasts from different cotton explants has been successful [28], the obtained protoplasts are very easily damaged during the transformation process, resulting in the failure of transformation. At present, there are no relevant reports on the transformation efficiency of cotton protoplasts. In this work, we used four enzyme solutions to deal with the properties of cotton polysaccharide and polyphenols. Through the transformation process, the key factors were explored to greatly improve the success rate of conversion. We systematically carried out gradient experiments one by one from callus culture, protoplast isolation, to transformation, and optimized the entire process.

In this study, we showed that when enzyme concentrations of 1.5% cellulase and 1.5% pectinase, and either 1.5% or 0.5% macerozyme and 0.5% hemicellulase were used, one can obtain increasingly stable protoplasts. When the plasmid concentration reaches 16 µg and the transformation time is controlled within 12–16 h, the best transformation efficiency can be obtained, and 35–45% of the protoplasts can be transformed.

Through the gradient exploration of the conditions for each step of isolating and transforming protoplasts, a highly efficient cotton callus protoplast isolation and transformation system was developed that can easily be used for protein subcellular localization studies. This was verified by transformation with GFP, YFP, and mCherry vectors, which demonstrated that the protoplasts isolated with this method can be used for downstream experimental research. The establishment of a transient gene expression system in cotton protoplasts will accelerate cotton functional genomics research and allow researchers to rapidly identify candidate functions for target genes. By optimizing the genetic transformation of cotton protoplasts, this research lays the foundation for high-throughput analysis of cotton genes.

## 4. Materials and Methods

### 4.1. Plant Materials and Growth Conditions

Seeds were cleaned by shaking in a tube with 70% ethanol for ~10 s, then discarding the alcohol. Hydrogen peroxide (H_2_O_2_, 30%) was then added and seeds were soaked for 2.5–3 h. Seeds were next washed by shaking in sterile water three times for ~30 s each. After sterilization and washing, seeds were incubated in 60 mL of sterile water for ~24 h, then placed in the culture room overnight until the seed coat burst. On a clean bench, seeds were then placed in Erlenmeyer flasks (four seeds per flask) containing seedling medium (MS medium with 3.3 g/L CaCl_2_). Seeds were incubated in the dark for 3 d, then incubated under a 16/8 h light/dark cycle for another 3 d. After seedlings grew, cotyledons and roots were removed and the remaining stems were placed into a Petri dish. A blade was used to cut both ends of each stem, then stems were cut into sections ~5–8 mm in length. Hypocotyl stem sections were then placed in the induction medium (MS medium with 3.3 g/L CaCl_2_, Fe^3+^ (2.78 g/L Fe_2_SO_4_), 30 g/L glucose, and trace vitamins (0.5 g/L VB1; 0.05 g/L VB6)) and sub-cultured until a callus was obtained.

### 4.2. Protoplast Isolation

For each sample, 1 g of callus tissue was weighed out and transferred with sterile tweezers to a 50 mL Erlenmeyer flask containing 7 mL of filtered sterilized enzyme solution. Flasks were sealed with parafilm and wrapped in aluminum foil, then shaken at 100 rpm for 3 h at room temperature (RT) in the dark. After enzymolysis, 5 mL W5 solution (2 mM Murashige and Skoog Medium with MES (MES) at pH 5.7 with 154 mM NaCl, 125 mM CaCl_2_, and 5 mM KCl) was added and the solution was gently shaken by hand to release the protoplasts. The solution was filtered with a 40 μm cell strainer and protoplasts were collected into a 50 mL centrifuge tube. The paper strips included on the nylon mesh surface of the cell strainer were then washed with W5 solution 3–5 times. Samples were centrifuged horizontally at 250× *g* and 4 °C for 5 min (with minimal acceleration and deceleration), and the supernatant was discarded. Protoplasts were resuspended in 2 mL of W5 solution. Samples were centrifuged at 200× *g* for 3 min at 4 °C (with minimum acceleration and deceleration), the supernatant was discarded, and protoplasts were resuspended in 80 μL mineral-modified glutamate (MMG) solution (4 mM MES at pH 5.7 containing 0.4 M mannitol and 15 mM MgCl_2_). Resuspended protoplasts were transferred to 1.5 mL centrifuge tubes.

### 4.3. Vector Construction and Plasmid Isolation

To verify whether the protoplasts isolated using this method could be used for plasmid transformation, we fused the conventional transient expression vector pCAMBIA1302-CaMV35S::*eGFP* to the membrane-localized *GhPIN1* gene, the *CPS4* gene (pCaMV35S::*CPS4*-*YFP*), a nuclear localization marker, a membrane-localized marker, or an endoplasmic reticulum (ER) localization marker. Plasmid extractions were performed with the Vazyme FastPure Plasmid Mini Kit (#DC201–01).

### 4.4. Protoplast Transfection

Polyethylene glycol (PEG)-mediated transfection was performed as described by [27] with some modifications. Briefly, PEG4000 (40% *w*/*v*) was freshly prepared in ddH_2_O containing 0.2 M mannitol and 100 mM CaCl_2_ at least 1 h before transformation to completely dissolve the PEG. Newly isolated protoplasts (100 μL) were mixed with 8–20 μg of plasmid DNA. For subcellular localization, 8–20 μg of total plasmid DNA was added to 110 μL of freshly prepared PEG4000. Tubes were inverted several times to mix the contents, then the mixture was incubated for 15–20 min in the dark. W5 solution (880 µL) was added to the tube and mixed well by inversion to stop the transformation process. Samples were then centrifuged at 250× *g* for 3 min at RT and the supernatant was removed. Protoplasts were gently resuspended in 2 mL of W5 solution. Plates were wrapped in aluminum foil and incubated at 23 °C for at least 12–16 h. Protoplasts were viewed under a microscope to determine their condition, with healthy cells appearing full and round. Transformation efficiency was determined by counting the number of GFP-fluorescing cells in the positive control using a fluorescence microscope.

### 4.5. Microscopy

A hemocytometer was used to determine the condition of extracted protoplasts. A cover glass was placed on the counting chamber, then protoplast suspension was dropped on one edge of the cover glass. After the protoplast suspension penetrated the gap between the cover glass and the counting plate and the counting chamber was filled, the number of protoplasts in the 16 middle squares were counted. Protoplast production was then calculated with the following formula:

protoplast # in 1 mL suspension = protoplast # in large squares × 10,000 × total volume (V) of protoplast suspension.

Protoplast viability was measured by mixing 20 μL of protoplast suspension with 2 μL 0.4% (*w*/*v*) trypan blue (pipetting to mix evenly) and incubating at RT for 3–5 min. A drop of the mixture was placed on a glass slide and covered with an 18 × 18 mm cover glass. The samples were viewed under a 10x objective lens and statistics (the total number of healthy and degraded protoplasts) were recorded for the four large squares in the corners of the cytometer.

Protoplast activity (%) was calculated as follows:

(number of protoplasts in 1–16 middle squares/16 total number of protoplasts in middle squares) × 100.

Average values were calculated from five fields of view; cells were excluded if they were too small, part of an agglomerated cell mass in which the number of cells could not be distinguished, or damaged by the transformation process.

## Figures and Tables

**Table 1:** The concentration of four enzymes used in different groups.

Group	Cellulase	Pectase	Macerozym	Hemicellulase
Group 1	0	1	1	1
Group 2	0.5	1	1	1
Group 3	1	1	1	1
Group 4	1.5	1	1	1
Group 5	1	0	1	1
Group 6	1	0.5	1	1
Group 7	1	1	1	1
Group 8	1	1.5	1	1
Group 9	1	1	0	1
Group 10	1	1	0.5	1
Group 11	1	1	1	1
Group 12	1	1	1.5	1
Group 13	1	1	1	0
Group 14	1	1	1	0.5
Group 15	1	1	1	1
Group 16	1	1	1	1.5

## Data Availability

Not applicable.

## Abbreviations

BAP	6-benzylaminopurine
NAA	1-naphthaleneacetic acid
IAA	indole-3-acetic acid
MS	Murashige and Skoog
MES	Murashige and Skoog Medium with MES
PEG	polyethylene glycol
